# Characteristics and treatment analysis of young acute coronary syndrome patients in a tertiary care hospital: A cross‐sectional retrospective study

**DOI:** 10.1002/hsr2.1141

**Published:** 2023-03-01

**Authors:** Jagannaathan Murugan, Jayanty Venkata Balasubramaniyan, Praveen Kumar Mathiyalagan, Yashwanth Ramesh, Meera Selvam, Chris Charley, Harini Muralidharan, Rishitha Venati, Indrani Devi Dhanasekaran, Muhasarparur Ganesan Rajanandh

**Affiliations:** ^1^ Department of Pharmacy Practice, Sri Ramachandra Faculty of Pharmacy, Sri Ramachandra Institute of Higher Education and Research Deemed to be University Porur, Chennai India; ^2^ Department of Cardiology Deemed to be University Porur, Chennai India; ^3^ Saveetha Medical College and Hospital, Saveetha Institute of Medical and Technical Sciences Deemed to be University Thandalam, Chennai India

**Keywords:** acute coronary syndrome, cardiovascular disease, risk factors, treatment, young

## Abstract

**Background and Aims:**

The prevalence of acute coronary syndrome (ACS) has been rising in the younger population worldwide. To fully comprehend the effects of the condition, it is crucial to examine the evolving characteristics and treatment options. The purpose of this study is to evaluate the characteristics and treatment analysis for young ACS patients in a tertiary care setting.

**Methods:**

This cross‐sectional, retrospective, single‐center study included a random sample of patients who had been hospitalized for ACS over the period of a year. We collected and analyzed data on risk factors, diagnoses, angiographic patterns, and potential treatments.

**Results:**

The study involved 198 young ACS patients in total. The majority of patients (57%) had no risk factors, and the majority of them (44%) had ST‐elevation myocardial infarction (STEMI) as their diagnosis. The most common type (48%) was single‐vessel disease (SVD). Statins and antiplatelet medications made up the majority of the patients’ nonsurgical treatments (88% and 87%, respectively). A statistically significant difference exists between young and older ACS patients with gender (*p* < 0.01). However, it is not clinically relevant.

**Conclusion:**

Men were the majority of young ACS patients, and STEMI, SVD were more prevalent. The majority of young ACS patients had no significant risk factors. To determine the risk factors of young ACS patients, a more thorough case–control study is critically needed.

## 1 INTRODUCTION

The leading global cause of morbidity and mortality is coronary artery disease (CAD). Acute coronary syndrome (ACS) and symptomatic CAD are uncommon among young adults under the age of 40, despite the fact that atherosclerosis, the primary cause of CAD, develops in the early stages of life.^1^

Patients with CAD under the age of 40 represent a special population subgroup that deserves attention. The majority of research has used age 45 as the cut‐off date to identify young people with CAD and myocardial infarction (MI).^2^ Unstable angina, non‐ST segment elevation myocardial infarction (NSTEMI), and ST‐segment elevation myocardial infarction (STEMI) are all included in the clinical spectrum of ischemic heart disease known as ACS. The prevalence of ACS among young people is quite modest when compared with the elderly.^3^

India has one of the highest rates of cardiovascular disease (CVD) compared with other countries.^4^ According to estimates, throughout the past several decades, the prevalence rates in India have varied between 1.6% and 7.4% for rural populations and between 1% and 13.2% for urban populations.^5^ Few studies have focused on the clinical presentation, treatment, angiographic profile, and prognosis of ACS in young patients because CAD occurs often in middle‐aged and elderly individuals.^4^, ^6^, ^7^, ^8^, ^9^, ^10^

According to the literature review, young people with ACS are more likely to have cardiovascular risk factors such as smoking, hyperlipidemia, obesity, and a family history of CAD.^3^, ^11^, ^12^, ^13^, ^14^ In India, particularly in urban areas, the prevalence of CVD risk factors has considerably increased during the past 25 years.^18^

Over the past 40 years, a considerable decline in CAD mortality rates has been attributed to the identification of risk factors as well as technological advancements in medicine. Thus, the purpose of this study is to evaluate the characteristics and analyses of the treatment of young patients with ACS at a tertiary care hospital in an urban location.

## 2 METHODS

### 2.1 Study design and site

A retrospective, cross‐sectional, single‐center study was carried out in the department of Cardiology, Sri Ramachandra Institute of Higher Education and Research (SRIHER), Porur, Chennai during July 2021 to July 2022.

### 2.2 Ethical considerations

The study was approved by the Human Institutional Ethical Committee of SRIHER, DU, Chennai, Tamil Nadu, India (CSP/21/JAN/89/43). Due to the retrospective nature of the study and the fact that it relied on previously collected data, informed consent was not obtained from the study participants. All data were anonymized and confidentiality was maintained throughout the study to protect the privacy of the participants. The study was conducted according to the National Ethical Guidelines for Biomedical and Health Research Involving Human Participants, Indian Council of Medical Research (ICMR), 2017.

### 2.3 Selection criteria

The study included all ACS patients who were hospitalized to the cardiology department for coronary angiography, regardless of gender. Patients who passed away before a diagnostic angiography, those with a history of past ACS or coronary revascularization, and more were excluded.

### 2.4 Study procedure

A specially designed data collection form was used to gather information on demographics, current smoking status, risk factors, clinical presentation, laboratory investigation, coronary angiography, treatment, and drug chart. Obesity is calculated using body mass index (BMI). Information on comorbid conditions was gathered from case files including past medical and medication histories. Laboratory results were also used to confirm in few cases.

### 2.5 Statistical analysis

The collected data were analyzed using Statistical Package for the Social Sciences (SPSS), version 21.0. Baseline characteristics were expressed as descriptive statistics. Categorical data were expressed as frequencies and percentages. The association between CAM usage and various variables like child's age and gender, parent's or caregivers’ education, occupation and socioeconomic status was assessed using the *χ* ^2^ test. A *p* value of less than 0.05 was considered statistically significant.

## 3 RESULTS

The study initially identified 500 consecutive patients who met the inclusion criteria. On further evaluation, 79 cases were excluded owing to insufficient documentation. The remaining 421 cases were included for this study. Of them, 198 were young ACS and their demographic details are given in Table 1. Most patients (57%) were devoid of the typical risk factors. However, as a secondary risk factor, diabetes, hypertension, and dyslipidemia contributed to approximately 17%, 13%, and 5% of cases, respectively. Furthermore, smoking, family history, and obesity represented only 3% of the total risk in our study, making it the least dangerous risk factor.

**Table 1:** Baseline characteristics of study participants.

Characters	*n*	%
Age (in years)		
15–29	21	11
30–40	177	89
Gender		
Male	166	84
Female	32	16
Risk Factors		
None	112	57
Diabetes	35	17
Hypertension	26	13
Dyslipidemia	11	5
Smoking	6	3
Family history	5	3
Obesity	3	2
Diagnosis		
STEMI	88	44
NSTEMI	64	32
UA	46	24
Angiographic profile		
SVD	95	48
LAD	42	21
LCX	17	9
RCA	36	18
DVD	43	22
LAD + LCX	21	11
LAD + RCA	13	7
LCX + RCA	9	4
TVD	34	17
Normal	26	13

Abbreviations: DVD, double vessel disease; LAD, left anterior descending artery; LCX, left circumflex artery; NSTEMI, non‐ST‐elevation myocardial infarction; RCA, right coronary artery; STEMI, ST‐elevation myocardial infarction; SVD, single vessel disease; TVD, triple vessel disease; UA, unstable angina.

The majority of patients (44%) presented with STEMI, and the coronary angiography profile revealed that single vessel disease (48%) was the most common result, followed by double vessel disease (22%) and triple vessel disease (17%). The left anterior descending artery was the single vascular disease artery that was most frequently impacted (21%). Additionally, 11% of cases of double vascular disease involved the left circumflex artery and left anterior descending artery.

The surgical treatments and medical management were listed in Table 2. A total of 58% of study patients underwent no surgical surgery, whereas 37% underwent percutaneous coronary intervention (PCI) and 5% underwent coronary artery bypass grafting. When pharmacological therapies were examined, statins (88%) such as atorvastatin and rosuvastatin were the most frequently utilized forms of therapy, followed by antiplatelet drugs (87%) such as aspirin, and clopidogrel. Anticoagulants (23%) such heparin and other anti‐anginals (15%) were used sparingly as a type of therapy in patients with ACS.

**Table 2:** Treatment patterns of study patients.

Treatment pattern	*n*	%
Surgical		
None	114	58
PCI	75	37
CABG	9	5
Medical management		
Statins	178	88
Anti‐platelets	175	87
Beta‐blockers	152	76
Aspirin	149	75
ACE inhibitors	105	55
Nitrates	90	47
Angiotensin receptor blockers	86	42
Calcium channel blockers	55	27
Anti‐coagulants	40	23
Other anti‐anginals	30	15

Abbreviations: ACE, angiotensin‐converting enzyme; CABG, coronary artery bypass grafting; PCI, percutaneous coronary intervention.

Table 3 shows the baseline characteristics of young (<40 years) and old (>40 years) ACS patients. Except for gender, which indicated a statistically significant difference between the two groups (*p* < 0.05 with a *χ* ^2^ value of 0.06), other variables were distributed equally between the two groups. Although gender difference is statistically significant between the groups, there is no clinically relevant difference because there are more male patients in both the groups.

**Table 3:** Association between characteristics and young ACS.

Characteristics of patients	ACS (<40 years)		ACS (>40 years)		*χ* ^2^ value	*p* value
	*N*	%	*N*	%		
Gender						
Male	166	84	183	82	0.06	<0.05
Female	32	16	40	18		
Risk Factors						
None	112	57	100	45	13.2	>0.05
Diabetes	35	17	48	22		
Hypertension	26	13	38	16		
Dyslipidemia	11	5	14	6		
Smoking	6	3	9	4		
Family history	5	3	8	4		
Obesity	3	2	6	3		
Diagnosis						
STEMI	88	44	97	43	0.29	>0.05
NSTEMI	64	32	72	32		
UA	46	24	54	24		
Angiographic profile						
SVD	95	48	102	46	9.6	>0.05
LAD	42	21	44	43		
LCX	17	9	20	20		
RCA	36	18	38	37		
DVD	43	22	49	22		
LAD + LCX	21	11	22	45		
LAD + RCA	13	7	16	33		
LCX + RCA	9	4	11	22		
TVD	34	17	40	18		
Normal	26	13	32	14		

Abbreviations: DVD, double vessel disease; LAD, left anterior descending artery; LCX, left circumflex artery; NSTEMI, non‐ST‐elevation myocardial infarction; RCA, right coronary artery; STEMI, ST‐elevation myocardial infarction; SVD, single vessel disease; TVD, triple vessel disease; UA, unstable angina.

## 4 DISCUSSION

The majority of ACS affects people over the age of 40. However, persons under the age of 40 have 5%–10% of MIs.^6^, ^15^, ^16^, ^17^, ^18^, ^19^, ^20^, ^21^, ^22^, ^23^, ^24^, ^25^, ^26^, ^27^, ^28^, ^29^, ^30^, ^31^, ^32^ The personal and societal costs of early coronary disease are significant, despite the fact that MIs in younger patients are typically associated with a good prognosis.^11^, ^17^, ^33^ Young people who have ACS experience early morbidity and mortality during what should be their most productive years of life.

CVD incidence and prevalence are significantly rising in developing countries like India, which results in rapid rise of global burden for the society. When compared with older patients' characteristics and treatment analysis, young patients had some significant differences in this study. To decrease mortality and morbidity, it is necessary to analyze the pattern of risk factors and the right treatment approach, even if they account for a relatively small portion of all cardiovascular disorders.

Unlike other studies (Esteban et al.,^22^ Hamm et al.,^23^ Schoenenberger et al.,^24^), this study compares the magnitude of various risk factors and treatment pattern that is present in the young acute coronary artery syndrome patients in a retrospective manner so as to audit tool for comparison of the established data with current or future practice. Similar to previous studies (Mirza et al.^25^), this study on analyzing the demographic variables found that the disease was more prevalent in male population less than 40 years of age which could imply that the age and gender plays a predominant role in modifying the disease progression in an indirect manner.

In contrast to a study conducted in Bangladesh by Patwary et al.,^26^ which found 73% of young ACS patients to be smokers, our research found that 3% of young ACS patients had smoking as a risk factor. Similarly, diabetes (42%) and hyperlipidemia (53%) were more common in their study. Only 5% of the patients in this study had dyslipidemia, while 13% of them had hypertension. While various risk variables were evident in the majority of studies, in this study 57% of young ACS patients had no risk factors.

STEMI was the most common clinical manifestation in this study and studies conducted by Tamrakar et al.,^27^ Wadkar et al.,^28^ and Sharma et al.,^29^ whereas NSTEMI and UA were more common in Hoo et al.,^30^ Iragavarapu et al.^31^ The most frequent angiographic procedure in this study is SVD, which is comparable to studies by Pathak et al.,^32^ Suresh et al.,^33^ and Al‐Koubaisy et al.^34^ The LAD contributed 21% of the total volume of the vessels implicated in younger age groups in Badran et al.^35^ and Ahmed Hussein et al.,^36^ studies, which are similar to this study. However, Kennelly et al.^37^ reported that the RCA was the most often affected artery. Similar to Jamil et al.^38^ and Sricharan et al.,^39^ TVD is uncommon among the study population.

The results of this study showed that statins (88%) were used to manage most of the patients, followed by antiplatelet drugs. Beta‐blockers, however, were the first preferred choice of medication in research by Davis et al.^40^ Aspirin (98%) and statins (81%) were the two most widely used medications for adults under the age of 45, according to Tungsubutra et al.^14^ study. The majority of patients in this study did not receive any surgical treatment. According to a study by Schoenenberger et al.,^24^ PCI was the treatment of choice for young ACS patients. Catheterization was reported in Davis et al.^40^

Our study exhibits potential limitations. First, it was a single‐center study with a small sample size, hence the results cannot be generalized to the community. Second, this is a retrospective study of data and hence confounding factors could not be taken into account. Moreover, most of the patients had no risk factors so the association between risk factors and young ACS could not be determined.

## 5 CONCLUSION

This study confirms that none of the known risk factors are associated with the development of the disease in young ACS patients, which prompts us to re‐evaluate the etiopathogenesis. Identifying this diseased cohort and implementing aggressive prevention measures in place to reduce risk factors will halt the disease progression. To identify the risk factors of young ACS patients and arrive at a conclusive diagnosis and course of treatment for the benefit of society, a more complete case–control research is required.

## AUTHOR CONTRIBUTIONS

**Jagannaathan Murugan**: Data curation; formal analysis; writing—review & editing. **Jayanty Venkata Balasubramaniyan**: Conceptualization; supervision; validation; writing—review & editing. **Praveen Kumar Mathiyalagan**: Data curation; formal analysis; writing—review & editing. **Yashwanth Ramesh**: Data curation; formal analysis; writing—review & editing. **Meera Selvam**: Data curation; formal analysis; writing—review & editing. **Chris Charley**: Data curation; formal analysis; writing—review & editing. **Harini Muralidharan**: Data curation; formal analysis; writing—review & editing. **Rishitha Venati**: Data curation; formal analysis; writing—review & editing. **Indrani Devi Dhanasekaran**: Data curation; formal analysis; writing—review & editing. **Muhasarparur Ganesan Rajanandh**: Conceptualization; supervision; validation; writing—original draft; writing—review & editing.

## CONFLICT OF INTEREST STATEMENT

The authors declare no conflict of interest.

## TRANSPARENCY STATEMENT

The lead author Muhasarparur Ganesan Rajanandh affirms that this manuscript is an honest, accurate, and transparent account of the study being reported; that no important aspects of the study have been omitted; and that any discrepancies from the study as planned (and, if relevant, registered) have been explained.

## Data Availability

All the authors have read and approved the final version of the manuscript. Corresponding author had full access to all of the data in this study and takes complete responsibility for the integrity of the data.
